# Primary Pericardial Mesothelioma: A Rare but Serious Consideration

**DOI:** 10.7759/cureus.19966

**Published:** 2021-11-28

**Authors:** Steffan Seal, Henry Simon

**Affiliations:** 1 General Medicine, Central Coast Local Health District, Gosford, AUS

**Keywords:** mesothelioma, asbestosis, cardiac tumour, mediastinum malignancy, pericardial mesothelioma

## Abstract

Primary pericardial mesothelioma (PPM) is an extremely rare malignancy with a very poor prognosis. It poses a diagnostic challenge given its often late and non-specific presentation. This report describes a 74-year-old man who presented with central pleuritic chest pain and mild breathlessness. The patient was febrile and mildly tachycardic with crepitations in the right lung base. Blood tests revealed raised inflammatory markers and chest X-ray showed no acute pathology.

Following admission, CT pulmonary angiogram showed a large left-sided mediastinal mass (approximately 110 x 70 x 85 mm) centered on the pericardium. Further post venous phase CT imaging identified possible myocardial invasion alongside suspicious liver nodules. Later, outpatient fluorodeoxyglucose (FDG) positron emission tomography (PET) imaging highlighted further FDG avid pleural and liver lesions. CT-guided biopsy of the pericardial lesion was undertaken, with histology and immunohistochemistry indicating epitheliod-type mesothelioma. A significant malignant pericardial effusion was also identified, which ultimately required pericardial window formation. Immunotherapy was commenced utilizing dual nivolumab and ipilimumab, a novel regime for the treatment of mesothelioma. Palliative radiotherapy to the pericardial lesion will also be performed.

Here, we demonstrate the diagnostic challenge of this vanishingly rare condition, which is usually diagnosed upon the development of associated complications. Early recognition gives the best chance of improved mortality, however, diagnosis requires a high index of clinical suspicion alongside prompt investigation, primarily involving cross-sectional imaging.

## Introduction

This case report describes a 74-year-old man who presented to the emergency department (ED) with non-specific chest pain. Initially diagnosed as a lower respiratory tract infection, further imaging and investigation in fact revealed primary pericardial mesothelioma, an extremely rare condition. This case highlights the non-specific and occult nature of presentation for this rare but serious condition, along with associated complications and management.

## Case presentation

Presentation

A 74-year-old retired boilermaker presented to ED complaining of central pleuritic chest pain and mild breathlessness. He reported lethargy but denied unexplained weight loss or night sweats. The patient had a background of stage IIIc sigmoid carcinoma, which was in remission, alongside pleural plaques and paroxysmal atrial fibrillation.

On examination, he appeared slightly pale. He exhibited a moderately increased work of breathing but was saturating normally on room air. The patient was febrile and tachycardic on the initial review. Coarse crackles were noted at the right base on auscultation and he was clinically euvolemic. Physical examination was otherwise unremarkable.

Investigations

Initial

A bedside ECG showed sinus rhythm with no ischaemic changes. Serial ECGs showed no dynamic changes. Blood tests obtained in ED revealed microcytic anemia, normal white cell count, normal renal function, mild hyponatremia (130 mEq/L), mild hypokalaemia (3.4 mmol/L), and a C-reactive protein of 150 mg/L. Initial troponin blood test was 16 ng/mL followed by a repeat at four hours of 14 ng/mL. *Imaging*

On initial assessment, a plain chest radiograph showed diffuse bilateral pleural plaques, which were stable when compared with previous imaging, and no other sign of significant pathology. Due to persistent pleuritic chest pain on a background of prior malignancy, a CT pulmonary angiogram (CTPA) was undertaken to rule out pulmonary embolus. A large left-sided mediastinal mass centered on the pericardium (measuring approximately 110 x 70 x 85 mm) was found. There was peripheral contrast enhancement with areas of hypoattenuation centrally.

**Figure 1:**
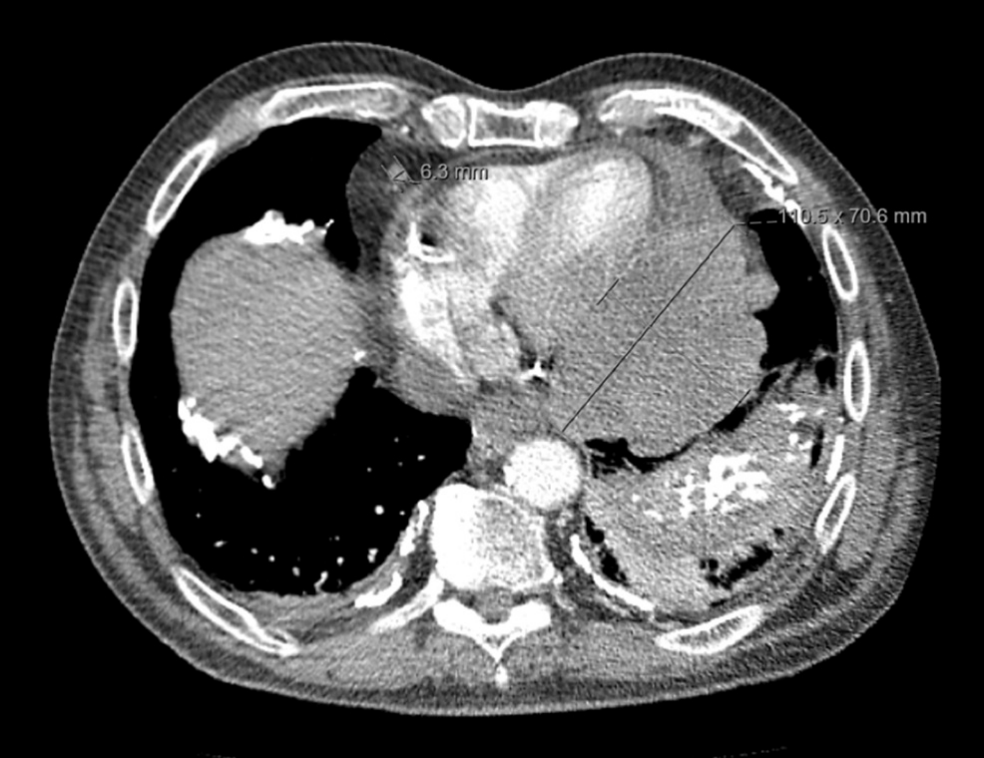
Cross-sectional CT showing pericardial mass measuring 110.5 x 70.6 mm Pleural plaques are present on the inferior aspect of the left lung.

**Figure 2:**
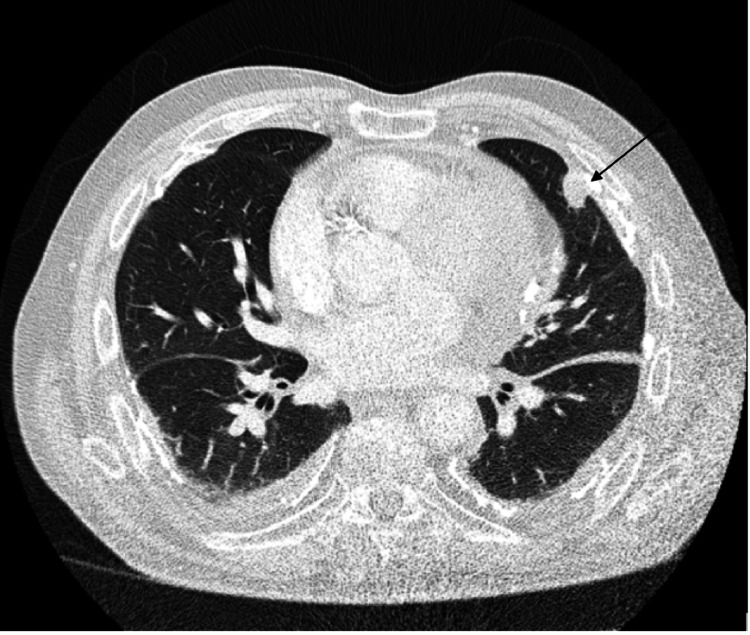
Axial slice CT scan showing lingular nodule that provided tissue diagnosis

**Figure 3:**
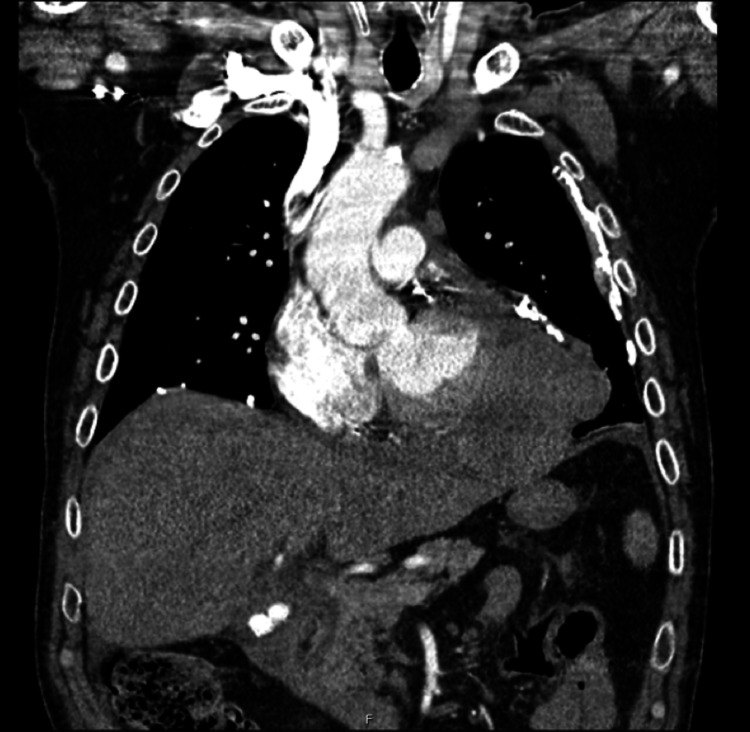
Coronal CT image showing pericardial mass with probable ventricular wall invasion

Enlarged para-aortic and sub-carinal lymph nodes increased suspicion for malignancy. In addition, a new hypodense lesion adjacent to the gallbladder fossa was noted, suggestive of metastatic disease. A moderate pericardial effusion was found, along with stable bilateral calcified pleural plaques. Arterial phase CT chest and portal venous phase CT abdomen and pelvis images were obtained to further characterize and stage the mass (Figures 1-3).

**Figure 4:**
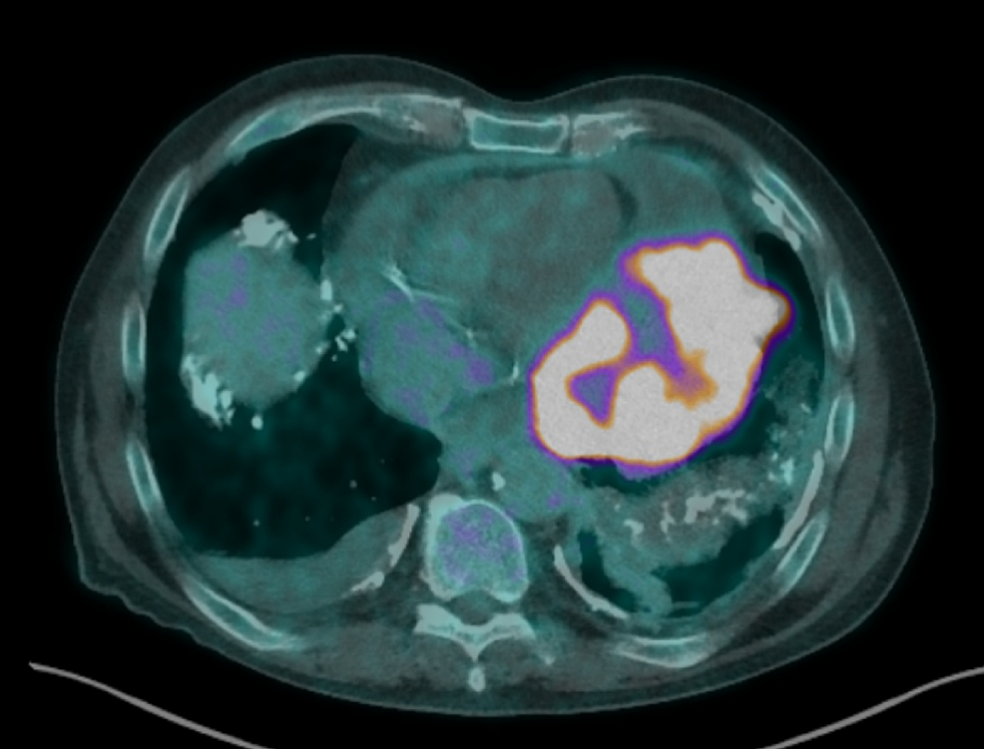
FDG-PET CT showing pericardial mass and effusion FDG: fluorodeoxyglucose; PET: positron emission tomography

Outpatient fluorodeoxyglucose (FDG) positron emission tomography (PET) scan showed an enlarging and intensely FDG avid mass at the left lower mediastinal border with a maximum standard uptake value (SUV) of 21.7 (Figure 4). A large pericardial effusion with a maximum of 4 cm depth was also identified (Figure 4). Further smaller pleural FDG avid masses were noted at the left anterior mid zone (SUV max 6.4), left anterior lower zone (SUV max 7.8), and left posteromedial lower zone (SUV max 5.3).

**Figure 5:**
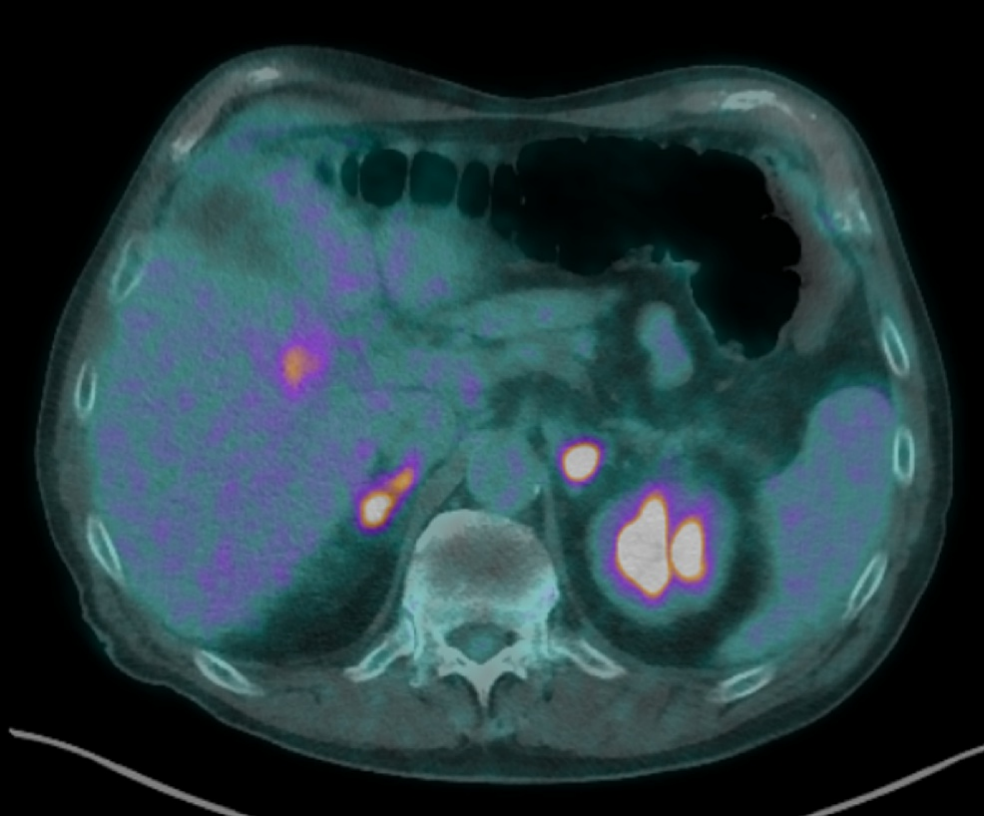
FDG-PET CT showing gallbladder fossa lesion FDG: fluorodeoxyglucose; PET: positron emission tomography

In the abdomen, further FDG avid masses were seen in segments five and seven of the liver, as well as FDG avid nodular thickening of both adrenal glands (Figure 5). Multiple suspicious lymph nodes in the abdomen, mediastinum, and supraclavicular regions were noted. No evidence of local recurrence of the previously treated colorectal carcinoma was seen.

Differential diagnosis

Initial diagnosis in ED was that of a right-sided pneumonia due to right lung crepitations alongside markedly raised inflammatory markers. Atypical acute coronary syndrome was also considered but ruled out following serial negative troponins alongside benign-appearing ECGs.

Following admission, initial diagnosis and management centered on community-acquired pneumonia. Later, due to ongoing chest pain, a CTPA was performed to rule out pulmonary embolus, which subsequently revealed a large mediastinal mass as the main significant finding. The radiological appearance of the mediastinal mass favored a primary thoracic malignancy. However, given the history of prior bowel malignancy, a metastatic process was still a differential. Possible primary malignancies included mesothelioma, lymphoma, or thymoma. Of note, there was a history of asbestos exposure alongside known asbestos-related lung plaques. CT-guided biopsy of a subpleural lingular nodule obtained three cores showing predominantly lesional tissue. Histopathology and immunohistochemistry findings were in keeping with epithelioid-type mesothelioma. The tumor cells were found to be positive for CK7, calretinin, D2-40, CK5/6, and WT1. After analysis of the imaging and histopathology, it was thought that the primary lesion was pericardial mesothelioma. 

Treatment

The patient was commenced on first-line therapy for primary mesothelioma in the form of immunotherapy agents, nivolumab and ipilimumab. Radiotherapy to the primary lesion was also being planned at the time of writing. Both immunotherapy and radiotherapy were undertaken with palliative intent.

PET-CT imaging showed a large malignant pericardial effusion, which was significantly increased in size from previous imaging. The patient was re-admitted to the hospital and drainage was achieved by way of pericardiocentesis and pericardial window without major complication.

## Discussion

Primary cardiac tumors are rare phenomena, with primary pericardial mesothelioma (PPM) being exceptionally rare overall. A large necropsy study of 500,000 cases found an incidence of <0.0022% [1]. These tumors are diagnostically challenging, with patients frequently presenting with non-specific symptoms. Onset is insidious with symptoms typically due to tumor-related sequelae. These include pericardial effusion with or without cardiac tamponade, constrictive pericarditis, and heart failure secondary to the neoplastic invasion of the myocardium. Approximately 200 cases have been reported in the literature, with only a quarter as antemortem diagnoses [2]. Appearances on CT and MRI are variable with potential for both cystic and solid appearances, pericardial effusion or pericardial thickening [3]. Here, the lesion demonstrated peripheral enhancement with central hypoattenuation in keeping with cystic changes, alongside a pericardial effusion. Additionally, there was a loss of flat plane appreciation between the mass and left ventricle in keeping with possible myocardial invasion.

Asbestos exposure is a well-known risk factor for pleural and peritoneal mesothelioma, for PPM this link is more ambiguous. One study analyzed PPM cases between 1993 and 2008 and found only three out of 14 total cases had previous asbestos exposure [4]. PPM tumors are known to present diffusely or as a localized mass. Three main histological types have been described - spindle cell, epithelioid, and mixed [5]. In this case, the histology almost certainly represents epithelioid-type disease.

Prognosis is generally very poor with median survival reportedly between two and six months [6,7]. This is likely because patients develop symptoms late in the disease course, by which time complete surgical resection is not possible. Moreover, PPM tends to respond poorly to both chemotherapy and radiotherapy.

There is no current standard treatment for PPM, with treatment for localized disease focusing on surgical debulking of the primary tumor. Treatment options are understandably limited in unresectable disease. Indeed, it has been noted that PPM patients are less likely to receive chemotherapy and have worse overall survival when compared with pleural mesothelioma patients [6]. However, a recent phase 3 study named CheckMate 743 demonstrated the efficacy of a combination of two monoclonal antibodies (nivolumab and ipilimumab) in the treatment of unresectable pleural malignant mesothelioma [8]. This combination of checkpoint inhibitors was most effective in treating epithelioid subtypes with a durable improvement in overall survival seen. This was most notable at two years with 41% overall survival in the ipilimumab and nivolumab arm, compared with 27% for the standard platinum-based chemotherapy arm. It is important to note that the study excluded patients with primary pericardial presentations and those with a prior malignancy without a three-year disease-free remission. Therefore, direct applicability to this case is difficult.

## Conclusions

Due to a paucity of cases, especially pre-mortem, studying this disease with clinically applicable findings is difficult. The late-stage at diagnosis and aggressive nature of mesothelioma mean that prognosis is poor, further hampering effective study. Here, we show how the diagnosis is commonly due to pathological sequelae, in this case, pleuritic chest pain likely secondary to a mediastinal mass. Although some improvements have emerged in the management of pleural mesothelioma, it remains to be seen how transferable these benefits will be to the treatment of PPM.
